# Splenic Vein Tumor Thrombosis in a Patient With an Oligometastatic Pancreatic Neuroendocrine Tumor: A Case Report and Literature Review

**DOI:** 10.7759/cureus.109218

**Published:** 2026-05-19

**Authors:** Andrew Francis, Tyler Cox, Adam Khader, Aamir Khan, Bryce S Hatfield, Ricardo Bello

**Affiliations:** 1 Surgical Oncology, Cancer Care NorthWest, Spokane, USA; 2 Surgical Oncology, Virginia Commonwealth University, Richmond, USA; 3 Pathology, Virginia Commonwealth University, Richmond, USA; 4 Surgery Transplantation, Virginia Commonwealth University School of Medicine, Richmond, USA; 5 Pathology, Virginia Commonwealth University Health System, Richmond, USA

**Keywords:** malignant tumor thrombus, optimal cytoreduction, primitive neuroectodermal tumor (pnet), tumor thrombus, vascular reconstruction

## Abstract

The presence of a splenic vein tumor thrombus in patients with well-differentiated pancreatic neuroendocrine tumors is rare and is seldom reported with histopathologic confirmation. Consequently, they pose distinct challenges in preoperative planning, particularly if sinistral portal hypertension is present. We present a patient with a well-differentiated pancreatic neuroendocrine tumor, splenic vein tumor venous thrombus, and oligometastatic disease to the liver, which was managed with upfront cytoreductive surgery and portal venous reconstruction. We achieved an R0 resection, and the patient had an uncomplicated clinical course. At the nine-month follow-up, he had no evidence of disease on surveillance imaging.

## Introduction

Pancreatic neuroendocrine tumors (PNETs) are a diverse group of rare tumors, representing 1-3% of all pancreatic neoplasms. They are diagnosed in approximately one per 100,000 people each year. However, this incidence has been increasing due to the widespread availability of imaging techniques, which have led to the incidental, early detection of many of these tumors [1,2].

Although PNETs are slow-growing, they often are diagnosed at an advanced stage and can be a source of considerable morbidity for affected individuals [3]. Despite the recent improvements in systemic therapies for advanced and metastatic PNETs, surgical intervention with cytoreduction remains the preferred treatment for selected patients with resectable metastases, as these patients can achieve longstanding symptomatic relief, prolonged progression-free survival, and longer overall survival after resection, in spite of a high risk of recurrence [4].

A rare characteristic of nonfunctional PNETs is the presence of a venous tumor thrombi, which is preceded by tumor venous invasion as opposed to venous occlusion. This feature has rarely been documented with histological confirmation in the literature [1,2,5-9]. We present a complex case of metastatic grade 2 pancreatic tail neuroendocrine tumor (NET) with oligometastatic disease to the liver and splenic vein tumor thrombus. Further, we highlight our multidisciplinary care and review the current literature on PNETS with tumor venous thrombosis.

## Case presentation

A 57-year-old African American male patient was referred to our health system for the management of a metastatic well-differentiated PNET, oligometastatic to the liver. His medical history included hypertension, well-controlled insulin-dependent diabetes mellitus, chronic kidney disease, grade D esophagitis, morbid obesity, and atrial flutter for which he was taking anticoagulation and antiarrhythmic medications. His family history was notable for a maternal aunt and uncle with pancreatic cancer. 

**Figure 1:**
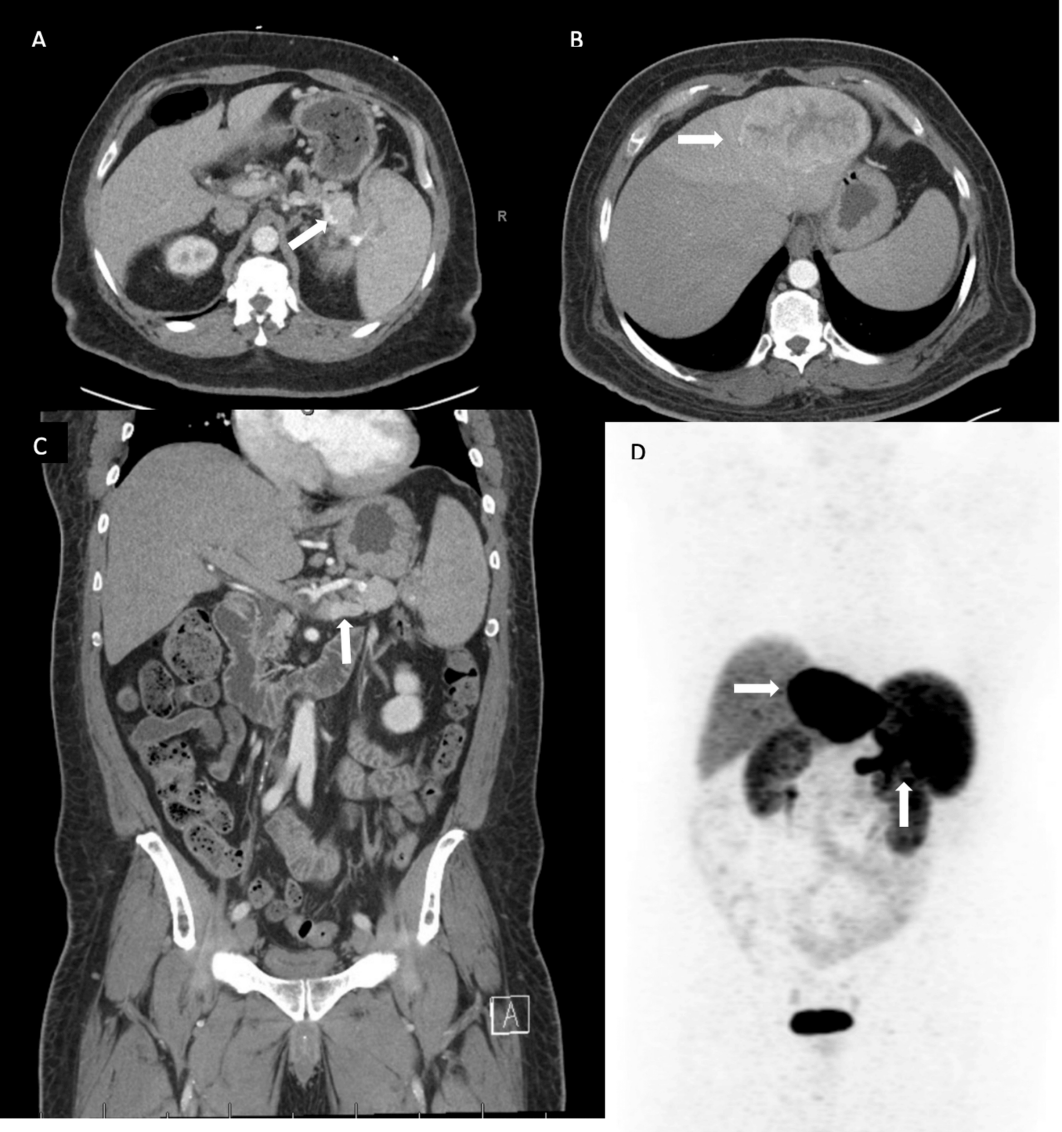
Imaging findings (A) Late arterial phase CT demonstrating pancreatic tail tumor (white arrow); (B) Early arterial phase CT showing liver segment 2 and 3 tumor (white arrow); (C) Late arterial phase CT showing possible splenic vein tumor thrombus (white arrow); (D) Cu-64 DOTATAE PET/CT depicting avidity of the splenic mass, extension into the spleen, and liver metastasis (white arrows).

He initially presented to an outside hospital with persistent, mild abdominal pain occurring in the morning that resolved spontaneously during the day. There was no associated GI dysfunction. A multiphase computed tomography (CT) scan of the abdomen and pelvis was performed, revealing a 3.6 x 2.2 cm ill-defined mass in the tail of the pancreas with 4.6 cm extension to the splenic hilum. There was no significant dilation of the pancreatic duct. Additionally, a heterogeneous enhancing lesion with central necrosis was found in the left hepatic lobe, involving segments 2 and 3, measuring 11.3 x 6.8 cm (Figure 1). 

The splenic vein appeared occluded, with multiple varices in the gastrohepatic ligament, perigastric area, and gastric region, suggestive of sinistral portal hypertension. The superior mesenteric vein (SMV), portal vein (PV), and hepatic veins remained patent. Tumor markers obtained at the outside institution, including alpha-fetoprotein (AFP), cancer antigen (CA)19-9, and carcinoembryonic antigen (CEA), were all within normal limits. 

**Figure 2:**
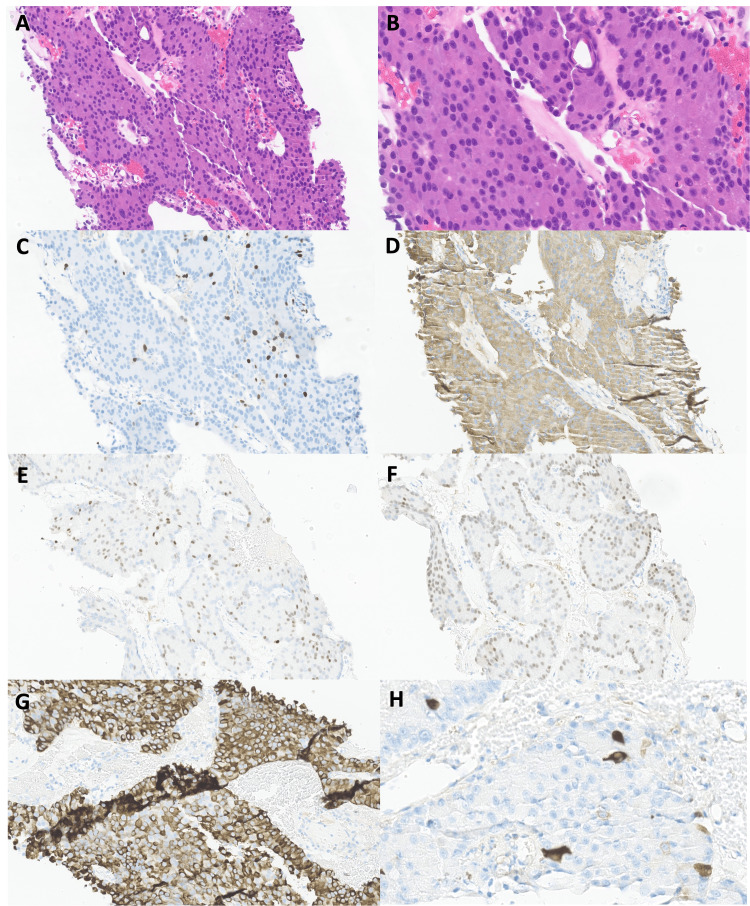
Pancreas biopsy (A, B) The tumor is arranged in solid sheets and cords with monotonous, bland nuclei and abundant eosinophilic cytoplasm (H&E, 20x and 40x, respectively); (C) Ki-67 staining shows a proliferation index up to 10% (Ki-67, 20x); (D) Positive staining for synaptophysin supporting neuroendocrine differentiation (synaptophysin, 20x); (E-H) Positive staining for CDX2, PAX8, CK19 (20x), and focal CK20 (40x), respectively.

A CT-guided biopsy of the liver mass was performed, and the biopsy results showed positivity for pankeratin, CK7, CK19, CK20, CDX2, PAX8, synaptophysin, and chromogranin, while being negative for hepatocyte-specific antigen (HSA), glypican 3, thyroid transcription factor 1 (TTF-1), and NKX3.1. The Ki-67 proliferation index indicated a focal area of increased activity of up to 10% (Figure 2). 

A diagnosis of a G2 well-differentiated NET was established. The patient was ultimately referred to our institution for further treatment.

Further laboratory testing and imaging were conducted at our institution. The chromogranin A level was 12,440 ng/mL. A Cu-64 DOTATATE positron emission tomography (PET)/CT scan (Figure 1D) showed increased radiotracer uptake in the left hepatic lobe mass with a standardized uptake value (SUV) of 76.4, and in the pancreatic tail mass with a SUV max of 59.8. Additionally, there was multifocal radiotracer uptake in lymph nodes along the greater curvature of the stomach, with an SUVmax of 30.5. No other signs of metastatic disease were detected.

The case was discussed during our multidisciplinary tumor board. It was recommended that the patient undergo upfront cytoreductive surgery, aiming for at least 70% cytoreduction of the tumor burden. Preoperatively, he received splenectomy immunizations and underwent esophagogastroduodenostomy, which revealed small esophageal varices measuring less than 5 mm, with no signs of bleeding, an irregular Z-line, large type 2 gastric varices, and portal hypertensive gastropathy. On the morning of the surgery, he underwent distal splenic embolization with 500-700 μm embospheres, which was followed by the planned open left lateral sectionectomy of the liver, distal pancreatectomy, splenectomy, and cholecystectomy.

**Figure 3:**
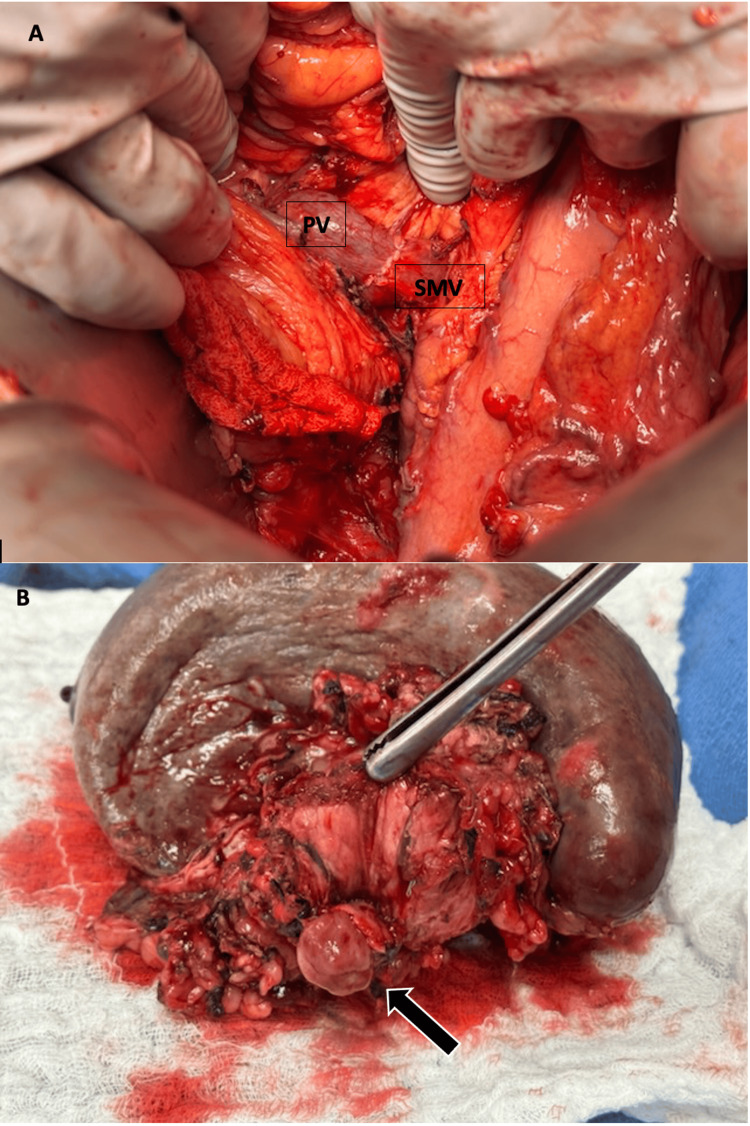
Intraoperative images (A) Intraoperative imaging of primary transverse venoplasty of the PV and SMV; (B) Tumor thrombus (black arrow) protruding from the splenic vein. PV: portal vein; SMV: superior mesenteric vein

Following laparotomy and intraoperative liver ultrasound, no evidence of occult metastatic disease was encountered. A left lateral sectionectomy of the liver and an open posterior radical antegrade modular pancreatosplenectomy were performed, which involved resecting the pancreatic tumor along with the distal pancreas, spleen, left adrenal gland, splenic flexure mesentery, and Gerota's fascia. A tumor thrombus was found on intraoperative ultrasound extending from the splenic vein into the PV/splenic vein confluence during resection. It did not appear to infiltrate the portal vein wall but extended intraluminally into the portal vein confluence. After isolating the proximal SMV and PV, a partial resection of the SMV and PV was performed at the level of the tumor thrombus origin, followed by vascular reconstruction through primary transverse venoplasty at the SMV-PV confluence (Figure 3).

Finally, multiple abnormal nodes from the greater and lesser omenta were excised in conjunction with a partial omentectomy. The patient had an unremarkable postoperative course and was discharged home on postoperative day eight.

**Figure 4:**
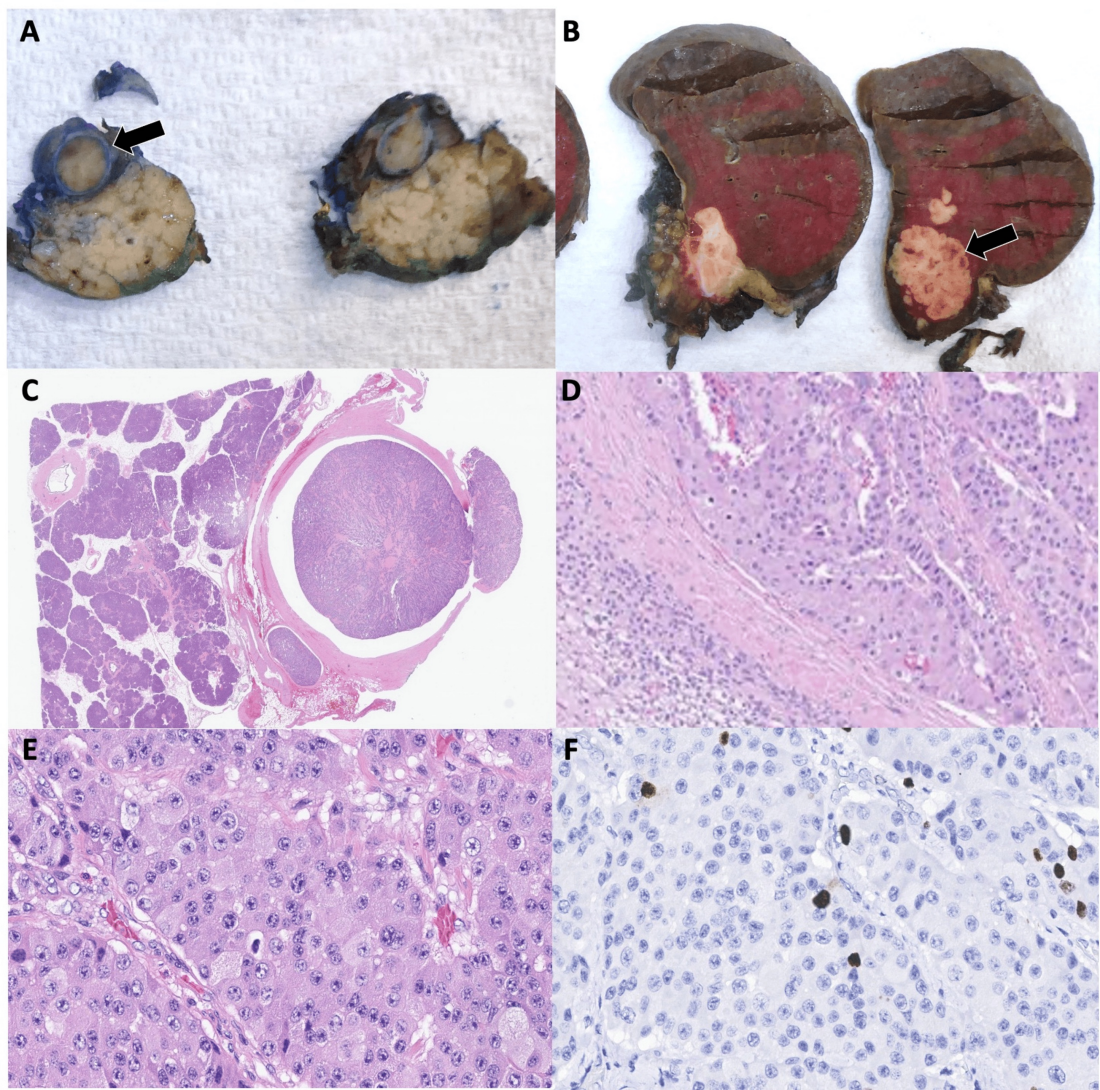
En bloc resection specimen and microscopic images (A) Gross image of the pancreas with splenic vein tumor thrombus (arrow); (B) Spleen with focus of metastatic carcinoma (arrow); (C) Low-power microscopic image of the pancreas with splenic vein tumor thrombus (H&E, 2x); (D) Low power image of metastatic neuroendocrine tumor involving the adrenal gland (H&E, 20x); (E) High-magnification image of D showing tumor cells arranged in sheets with monotonous nuclei, prominent nucleoli, and abundant eosinophilic cytoplasm (H&E, 40x); (F) Immunohistochemical staining for Ki-67 shows a proliferation rate between 3% and 20% (Ki-67, 40x).

On gross examination, the pancreatic tumor measured 9 cm and involved the pancreatic body and tail, spleen, and adrenal gland. The left lateral segmentectomy specimen contained a metastatic NET up to 10 cm in size and was located 1.2 cm from the parenchymal margin. On microscopic examination, the NET was arranged in an organoid growth pattern and consisted of small and monotonous round cells with coarse chromatin and eosinophilic, granular cytoplasm. Immunohistochemical staining for Ki-67 showed a 3-20% proliferation consistent with a grade 2 well-differentiated NET (Figure 4). 

Lymphovascular and perineural invasion were present. The tumor directly invaded the spleen, adrenal gland, and splenic vein. All margins were negative. Metastatic tumor involvement was found in three of eight regional lymph nodes and in two non-regional lymph nodes from the lesser curvature. Final pathologic stage was pT4N1M1c (American Joint Committee on Cancer (AJCC) Cancer Staging System, version 9 [10]). 

The patient did not experience any 30-day postoperative complications, nor did he require any increase in his insulin dosage. After a multidisciplinary review, he was offered clinical trial participation for adjuvant therapy but declined due to personal reasons. Through shared decision-making, we elected for quarterly imaging surveillance. At the nine-month follow-up, systemic imaging with CT chest, abdomen, and pelvis showed no evidence of disease recurrence.

## Discussion

We presented a case of a well-differentiated PNET with oligometastatic disease of the liver and a tumor thrombus in the splenic vein extending to the SMV/PV confluence. The occurrence of splenic venous tumor thrombus in this context is uncommon and has only been documented in a few case reports and small case series (Table 1) [1,2,4,5,7]. 

**Table 1:** Reports of splenic venous abnormalities from pancreatic neuroendocrine tumors NR: not reported; Y: yes;  N: no; PNI: perineural invasion; LVI: lymphovascular invasion

Author	Year of Publication	Sum of pts	Presenting symptoms	Primary location	Size (cm)	Metastatic disease or high-risk features	Surgical treatment	Histopathologic confirmation of tumor thrombus	Stage
Bok et al. [4]	1984	4	NR	3 Pancreatic body 1 Pancreatic head	NR	Y, Liver metastases (2)	NR	N	NR
Balachandran et al. [2]	2012	21	NR	NR	NR	NR	Seven underwent (unknown) surgical intervention	Y (Seven had documented microscopic tumor thrombosis)	NR
Rodriguez et al. [1]	2014	1	Longstanding left subcostal pain	Pancreatic tail	3.2	Y, PNI and LVI	Open distal pancreatectomy and splenectomy	Y	T2N0M0
Miyata et al. [5]	2020	1	Upper abdominal pain	Pancreatic body	2.5	NR	Open total pancreatectomy	Y	T2N1M0
Garcia Soria et al. [7]	2023	1	GI bleed	Pancreatic body and tail	NR	N	Distal pancreatectomy and splenoportal axis thrombectomy with vascular splenic resection (patient had prior splenectomy)	N	N

Although both pancreatic adenocarcinoma and PNETs have a propensity for venous occlusion, arterial involvement, lymphadenopathy, and liver metastasis, PNETs exhibit distinctive patterns of local invasion, including intraductal growth and venous tumor thrombosis. The incidence of venous tumor thrombus in nonfunctional PNETs is unknown, and most reported cases of venous tumor thrombus in PNETs involve the portal vein [2]. 

In an early observational study, Bok et al. reviewed 76 patients with pancreatic “islet cell tumors” [4]. Only 10 patients had angiographic evidence of venous abnormalities. Of these 10 patients, two had reported splenic venous occlusion, while two others had evidence of splenic venous encasement. No patients with intravenous tumor extension on imaging had splenic vein abnormalities. Additionally, only two patients underwent successful resection with histological confirmation, and neither has splenic vein involvement. As such, it remains unclear whether their angiographic findings were substantive, as no histologic confirmation was provided. 

Balachandran et al. published a retrospective review of 88 patients over four years with CT evidence of venous tumor thrombi with pathologically confirmed nonfunctional PNET [2]. They reported 21 cases of splenic thrombus, 15 alone and six with either PV confluence or inferior mesenteric vein involvement. Despite being the largest review on venous involvement, it is unclear how many patients had pathologically confirmed splenic vein thrombosis. Many patients either did not undergo surgical intervention or had initial discordant findings on the CT imaging, and they only reported patients with histologically confirmed microscopic splenic tumor thrombi. Importantly, it was noted that inaccurate reporting of this finding could result in incomplete surgical resection or missed opportunities for tailored surgical planning. They determined that CT's sensitivity for detecting venous tumor thrombus, gross or microscopic, was 52.4%, and its specificity was 100%. Further, the presence of venous tumor thrombi was associated with significantly larger tumors (5.8 cm vs 4.5 cm, p =0.005). 

Unfortunately, neither of these retrospective reviews consistently reported histologically confirmed splenic vein involvement, a common drawback in many reported cases. To date, we are aware of only two other case reports describing splenic vein tumor thrombosis with pathologic confirmation. Rodriguez et al. reported a patient with a biopsy-proven 3.3 cm nonfunctional PNET located in the tail of the pancreas with a concurrent 1.8 cm splenic tumor thrombus that extended 5 cm distally [1]. They performed an open distal pancreatectomy and splenectomy. Histological analysis of the thrombus confirmed the presence of indeterminate PNET. Similar to our case, both patients had evidence of sinistral portal hypertension; however, splenic venous tumor thrombosis was not discovered on preoperative imaging nor documented during operative intervention. Further, the patient's outcome was not reported. Miyata et al. reported a nonfunctional PNET associated with pancreatic duct ingrowth and splenic vein tumor thrombus [5]. Their patient underwent a total pancreatectomy due to positive margins along the duct. Final pathology confirmed a grade 2 well-differentiated PNET with evidence of regional nodal metastases. During the examination, the tumor was found to protrude into the splenic vein. Akin to our experience, this patient had no complications, and there was no recurrence of the tumor over a period of 13 months, despite the absence of adjuvant therapy. 

Despite some similarities, we report the only case involving resection of oligometastatic disease and reconstruction of the PV-SMV confluence, a procedure previously considered a contraindication for resection in some series [2]. Liver resection for neuroendocrine metastases can lead to long-term survival and better disease control, particularly in those without high-risk features such as distant lymph node metastasis, extrahepatic metastasis, diffuse peritoneal metastasis, right cardiac dysfunction, or well-differentiated grade 3 histology or neuroendocrine carcinoma. However, potentially curative surgery is achievable in only 10-25% of patients with liver metastases [3,4].

In addition, in the modern era, most patients with PNETs undergo advanced imaging with DOTATATE PET, for which Gallium-68 was approved in 2016, and newer agents such as Cu-64 were approved in 2020 [11]. While they have increased sensitivity for detecting NETs compared to conventional imaging, it is unclear whether these imaging studies would similarly increase the detection of tumor venous thrombi. Nevertheless, the presence of sinistral hypertension should raise clinical suspicion of splenic vein involvement. We recommend preoperative splenic artery embolization to decompress variceal disease from sinistral portal hypertension.

Finally, the prognostic importance of splenic vein tumor thrombus remains unknown due to its low incidence. It is also unclear whether its presence worsens overall or disease-free survival in the setting of resectable oligometastatic disease. Conversely, splenic vein tumor thrombi are common in patients with pancreatic ductal adenocarcinoma, being observed in up to 32% of patients undergoing distal pancreatectomy, and in one study have been found to be an independent risk factor for overall survival [12].

## Conclusions

To our knowledge, we have presented one of the few cases of a well-differentiated pancreatic neuroendocrine tumor with documented histologically confirmed splenic vein tumor thrombus. Further, this is the only case with oligometastatic disease to the liver managed with upfront cytoreductive surgery and complex portal venous reconstruction. Venous tumor thrombus presents a challenge in preoperative planning for patients with well-differentiated PNETs, especially in the setting of sinistral portal hypertension, and requires diligent preoperative assessment and preparation. Pathological confirmation is essential for a definitive diagnosis of tumor venous thrombi. 

## References

[1] Rodriguez RA, Overton H, Morris KT (2014). Pancreatic neuroendocrine tumor with splenic vein tumor thrombus: a case report. Int J Surg Case Rep.

[2] Balachandran A, Tamm EP, Bhosale PR, Katz MH, Fleming JB, Yao JC, Charnsangavej C (2012). Venous tumor thrombus in nonfunctional pancreatic neuroendocrine tumors. AJR Am J Roentgenol.

[3] Morgan RE, Pommier SJ, Pommier RF (2018). Expanded criteria for debulking of liver metastasis also apply to pancreatic neuroendocrine tumors. Surgery.

[4] Bok EJ, Cho KJ, Williams DM, Brady TM, Weiss CA, Forrest ME (1984). Venous involvement in islet cell tumors of the pancreas. AJR Am J Roentgenol.

[5] Miyata T, Takamura H, Kin R (2020). Pancreatic neuroendocrine tumor featuring growth into the main pancreatic duct and tumor thrombus within the splenic vein: a case report. J Surg Case Rep.

[6] Ait-Ali A, Sall I, El-Kaoui H (2010). Medial pancreatectomy for a neuroendocrine tumor invading the splenic artery and vein. JOP.

[7] García Soria A, Brotons Brotons A, Girona Torres E (2023). Gastric variceal bleeding as a form of presentation of a pancreatic neuroendocrine tumor. Rev Esp Enferm Dig.

[8] Nguyen BD (2005). Pancreatic neuroendocrine tumor with portal vein tumor thrombus: PET demonstration. Clin Nucl Med.

[9] Bello RJ, Clarke CN (2024). Evolution of surgical management and outcomes of neuroendocrine tumor liver metastases. Ann Surg Oncol.

[10] Rindi G, Dasari A, Hope TA (2024). Neuroendocrine tumors of the pancreas. AJCC Cancer Staging System: Neuroendocrine Tumors of the Pancreas (Version 9 of the AJCC Cancer Staging System).

[11] Jha A, Patel M, Carrasquillo JA (2026). Choice Is good at times: the emergence of [(64)Cu]Cu-DOTATATE-based somatostatin receptor imaging in the era of [(68)Ga]Ga-DOTATATE. J Nucl Med.

[12] Jeune F, Collard M, Augustin J (2024). Splenic vein tumor thrombosis is a major prognostic factor in distal pancreatic adenocarcinoma. Surgery.

